# Supplementary material for: Are carbon emissions trading and green financial instruments synergistic? -Comprehensive quantitative research based on content analysis
Source: PLoS One. 2024 Mar 7;19(3):e0298601. doi: 10.1371/journal.pone.0298601 (PMC10919601; doi:10.1371/journal.pone.0298601)
Supplement: S1 Data — (ZIP) [file pone.0298601.s001.zip › Data/地级市Z_F工作报告.zip!/║╙▒▒/╠╞╔╜╩╨/└┤╫╘╣1⁄2╓┌║┼í╛┬φ┐╦╩2╛▌═°í┐_2011.docx]

一、2010年工作回顾

刚刚过去的一年，是经济社会发展环境极为复杂的一年。面对严峻挑战和繁重的发展任务，全市人民在市委的坚强领导下，坚决落实中央和省一系列重大决策部署，以科学发展示范区建设为总揽，把推进经济平稳较快发展与加快经济发展方式转变有机统一起来，坚定信心，迎难而上，扎实苦干，较好地完成了市十三届人大三次会议确定的目标任务，各项事业取得了新的成绩。

(一)全力推动经济平稳较快发展，综合实力跨上新台阶。预计全市完成地区生产总值4300亿元，增长13%;全部财政收入439亿元，其中一般预算收入196亿元，分别增长6.2%和15.5%。组织实施千个项目持续攻坚行动，预计全社会固定资产投资2616亿元、增长20%左右，张唐铁路等402个项目开工，首钢京唐钢铁一期等200个项目投产或投入使用，承唐高速二期等一批重大基础设施项目竣工。狠抓工业运行调节，力促工业经济提质增效，规模以上工业完成增加值2050亿元，增长16%。强化政策引导，拉动城乡消费，全年社会消费品零售总额1100亿元，增长17.7%。

(二)着力调整产业结构，转变经济发展方式取得新进展。推动信息化工业化融合发展，组织开展“三百计划”和对标行动，实施技术改造项目667项。钢铁企业整合重组扎实推进，前五强企业生产集中度达到66%，精品钢比重达60%。丰润中国动车城等装备制造产业园区建设取得重要进展，高速铁路客车制造、冶金矿山机械、工程机械三大装备制造产业链不断延伸，节能环保、新能源和电子信息等新兴产业加速发展。被列入首批国家创新型试点城市和国家节能与新能源汽车示范推广试点城市，高新技术产业园区晋升为国家级高新区，国家高速动车组高新技术产业化基地落户我市。工业聚集区建设步伐加快，具备一定开发规模的工业聚集区达到20个，其中4个年主营业务收入超过100亿元。服务业规模和水平进一步提升，海港物流等五个省级物流聚集区建设扎实推进，乐购、沃尔玛等一批现代流通业态项目相继开工。持续开展节能减排攻坚行动，预计全年削减二氧化硫6.64万吨、化学需氧量1.3万吨、节能181万吨标准煤，可全面完成2010年和“十一五”节能减排目标任务。在全国城市环境综合整治定量考核中，我市由三年前的全省末位跃升为第二位。

(三)加快推进唐山湾“四点一带”开发建设，生产力布局得到新优化。预计“四点一带”区域完成地区生产总值1350亿元，增长13%，占全市经济总量的32%。曹妃甸新区完成投资1000亿元。曹妃甸港区矿石码头二期建成，煤炭码头二期等港口建设进展顺利，LNG码头、京唐港区20万吨级航道工程开工。唐山港全年吞吐量达到2.4亿吨，成为国内第10个吞吐量突破2亿吨的大港。曹妃甸新增造地28.3平方公里，海滨大道竣工通车，供水、供电、污水处理等基础设施建成投入使用。产业聚集步伐加快，“四点一带”区域实施重点产业项目375项，总投资6172亿元，完成投资528.5亿元。冀东风电装备研发制造、中视中科激光显示产业基地一期等一批项目开工建设。中日唐山曹妃甸生态工业园建设取得积极进展，8个项目入园开工。唐山湾生态城城市基础框架初步形成。

(四)深入推进城镇面貌三年大变样，新型城镇化迈出新步伐。全市城镇建设完成投资900亿元，城镇面貌三年大变样目标较好完成，在全省三年大变样评比考核中名列第一。成功申办2016年世界园艺博览会，成为国内首个地级承办城市。“四城一河三岛”开发建设取得重要进展。南湖生态城迎宾大道、休闲美食广场等一批重点项目相继完工，生态景观全面提升，西北片区改造回迁安置房建设进展顺利;凤凰新城市政基础设施不断完善，唐山一中新校投入使用，新青少年宫等一批项目加紧建设;三女河机场正式通航，空港城起步区建设全面启动;唐山湾生态城科教城区、企业服务区、滨海旅游休闲区等功能区建设进展顺利;57公里环城水系通水通航，华北水城框架初步形成;唐山湾国际旅游岛总体规划编制完成，一批项目陆续启动。旧城改造稳步推进，启动实施新华楼、新华文化广场等重点区域改造和大体量公建项目建设，拆除震后危旧平房282万平方米、开工安置住房550万平方米，市中心区19个城中村改造进展顺利，完成既有建筑节能改造和热计量改造800万平方米。新火车站、老唐山风情小镇等十大标志性精品建筑开工建设。城市环境综合整治和绿化攻坚行动取得明显成效，被评为全国绿化模范城市。县城扩容和小城镇建设加速推进，城镇化率达到54.9%，同比提高1.5个百分点。

(五)坚持统筹城乡发展，新农村建设呈现新气象。制定实施《推进统筹城乡发展，加快实现城乡等值化的若干政策》，省级统筹城乡发展试点市建设取得积极进展。各项惠农政策全面落实，农村公益事业“一事一议”财政奖补比例、资金额度居全省首位。粮食生产再获丰收，总产达311.7万吨，增长2.6%，连续7年实现增产。深入实施设施农业、规模养殖、专业合作社建设等十大工程，集中建设了一批现代农业示范园区，谋划建设农业产业化带动项目115个，农业产业化经营率达到64%，同比提高1个百分点。农村劳动力向非农产业转移15万人，非农收入占农民人均纯收入比重达到70%以上。文明生态村和科学发展示范村建设扎实推进，硬化农村道路1350公里，垃圾集中清运处理达标村(居)达到总村(居)数的95%。农村新民居建设加速推进，300个省级示范村全部开工，农村面貌和人居环境有了新的提升。

(六)不断深化改革、扩大开放，经济社会发展增添新动力。市县两级政府机构改革全面完成。县乡财政体制改革取得积极进展。文化体制改革主要任务基本完成。医药卫生体制改革稳步实施，科技、教育、社会管理等领域改革不断深化。地方金融运行质量显著提高，光大银行、华夏银行等17家金融机构入驻唐山。民营经济快速发展，增加值占全市生产总值比重达到67.2%。对外开放层次和水平实现新提升。成功举办了首届中国•曹妃甸临港产业国际投资贸易洽谈会、第十三届陶博会等重大招商推介活动。预计全年实际利用外资8.8亿美元，完成进出口总额72亿美元，分别增长10.4%和18%;对外投资总额1.1亿美元，增长87.2%。对内交流与合作取得新成效，实际引资266亿元，增长11.3%，区域合作的深度和广度进一步拓展。

(七)着力保障和改善民生，各项社会事业实现新发展。加大民生领域投入，为群众办的20件实事全部完成。城镇居民人均可支配收入和农民人均纯收入达到19515元和8310元，分别增长8.1%和12%。全市城镇新增就业8.6万人，城镇登记失业率4.06%，低于省达控制目标0.44个百分点，“零就业家庭”动态归零。城镇养老、医疗、失业、工伤、生育保险参保率和保障水平均居全省前列，城乡低保标准进一步提高。新型农村养老保险参保人数达到121万人，29.9万农民领取养老金。新开工建设经济适用住房3000套、公共租赁住房500套、廉租住房3204套。各项社会事业全面进步。义务教育均衡发展，职业教育规模不断扩大，唐山工业职业技术学院曹妃甸新校区建设进展顺利，河北理工大学、华北煤炭医学院合并组建河北联合大学，获博士学位授予权立项建设，唐山学院北校区二期和东校区扩建投入使用。文化事业蓬勃发展，电影《唐山大地震》全国热播，6部作品入选全国“五个一工程奖”，河北文化创意产业园等重点文化项目加快推进，传媒大厦开工，成功举办第七届中国评剧艺术节。新唐山人文精神进一步弘扬，我市赴青海玉树抗震救灾医疗抢险队被党中央、国务院和中央军委授予全国抗震救灾英雄集体荣誉称号。圆满完成援建四川地震灾区南坝镇恢复重建任务，唐山援建队伍获得全国“五一”劳动奖状。体育事业发展水平不断提高，唐山籍运动员在第十六届广州亚运会上创造了3金、6银的国际综合体育赛事最好成绩。“健康唐山、幸福人民”行动向纵深推进，免费为全市190万45岁以上农民和城镇无业居民进行了体检;城乡卫生服务体系进一步完善，标准化卫生室建设率达到80%;凤凰新城新工人医院开工建设。爱国卫生运动广泛开展，食品药品安全专项整治取得实效。全面贯彻计划生育基本国策，人口自然增长率控制在3.02‰。民族宗教、外事侨务、双拥、防震减灾、妇女儿童、老龄、气象、档案、人防、民兵预备役和国防动员等各项事业全面发展。深入推进社会矛盾化解、社会管理创新、公正廉洁执法，正确处理人民内部矛盾，积极妥善化解各类矛盾纠纷，强化社会治安综合治理，狠抓安全生产工作，稳定和谐的社会局面得到进一步巩固。

(八)切实加强政府自身建设，行政效能和服务水平有了新提高。按照中央、省和市委统一部署，认真开展“创先争优”活动，在党员干部中形成了创科学发展之先、争科学发展之优的生动局面。巩固“作风建设年”活动成果，深入推进干部作风建设，扎实开展“万名干部下基层、促进经济发展方式转变”活动。政府规章、规范性文件和行政审批事项清理规范工作进一步深化。城乡一体化服务管理信息系统实现城乡覆盖，全天候为市民提供优质高效服务。规范行政行为，严格行政执法。加强政风和廉政建设，推行权力运行监控模式，实施行政机关“晒权评议”，惩治和预防腐败体系建设取得新成效。自觉接受人民代表大会及其常委会的依法监督，重视发挥人民政协的政治协商、民主监督和参政议政作用，承办人大代表建议和政协委员提案676件，按时办结率100%，代表、委员满意率97%，促进了政府工作水平的提高。

各位代表，过去的一年是我市经济社会发展取得令人鼓舞新成绩的一年，为“十一五”划上了圆满句号，为“十二五”开好局、起好步打下了坚实基础。这是党中央国务院、省委省政府和市委正确领导的结果，是全市人民众志成城、顽强拼搏、共克时艰的结果。在此，我代表市政府，向全市工人、农民、知识分子、各级干部和广大建设者，向人大代表、政协委员，向各民主党派、工商联、无党派人士和人民团体，向驻唐部队、武警官兵、公安政法干警以及关心支持唐山发展的各界朋友，表示崇高的敬意和衷心的感谢!

总结过去的一年，我们也清醒地认识到，我市经济社会发展中仍存在着诸多亟待解决的问题。主要是：经济结构性矛盾仍然突出，工业结构偏重、服务业发展滞后，自主创新能力不强、低端产品比重大;经济增长方式仍较为粗放，资源环境约束进一步强化，节能减排任务艰巨，钢铁等支柱产业抵御市场风险能力还不够强，经济运行质量和效益有待提高;城镇化进程与经济发展水平还不相适应，城乡二元结构矛盾依然突出，城市聚集产业能力、城市功能、内涵和品位有待进一步提升;对外开放水平偏低;经济社会协调发展的任务艰巨，与人民群众的愿望和要求还有一定差距;财政收入支出压力加大，保障和改善民生任务繁重;政府自身建设有待进一步加强，一些政府工作人员的工作理念、方式、作风还不能适应新形势的要求和人民群众的期望。对这些问题，我们必须以对党和人民高度负责的态度，采取更加有力的措施，认真加以解决。

二、2011年主要任务

2011年是“十二五”开局之年。做好今年工作，具有特殊重要意义。展望2011年，机遇与挑战并存。从机遇看，国际金融危机催生新的科技革命，我国发展长期向好趋势没有改变，国家京津冀地区区域发展规划即将出台，河北沿海地区开发即将纳入国家发展战略，省委、省政府围绕打造沿海经济带出台一系列扶持政策。就我市来讲，伴随着改革开放的不断深化，唐山经济社会发展取得了巨大成就，特别是以曹妃甸为龙头的沿海经济隆起带已具雏形，城市功能不断完善、环境形象大为改观，一批重大项目启动实施，为唐山长远发展积蓄了强大后劲。从挑战看，外部环境极其复杂、世界经济复苏进程艰难曲折，国内经济发展中的短期问题和长期问题交织、结构性问题和体制性矛盾叠加，通胀预期增强，金融环境趋紧;唐山作为资源型城市，转变经济发展方式、调整经济结构任务艰巨，国家宏观政策调整对我市经济传导快、影响大;同时，我们还面临着区域竞争日益激烈带来的巨大压力。对当前形势，我们一定要审时度势，保持清醒头脑，既要增强责任意识、机遇意识，充分看到有利条件和积极因素，又要增强忧患意识、危机意识，充分估计各种困难和不利因素，牢牢把握工作的主动权。

围绕贯彻落实中央和省总体部署和要求，根据市委八届七次全会精神，今年全市国民经济和社会发展总体思路是：认真贯彻党的十七大和十七届五中全会、省委七届六次全会、市委八届七次全会精神，以科学发展为主题，以加快转变经济发展方式为主线，坚持把“稳增长、调结构、控物价、惠民生”作为主攻方向和着力点，着力调整产业结构，推动我市由制造业大市向制造业强市转变;着力发展沿海经济，推动经济发展由内陆资源型向沿海开放型转变;着力加强科技创新，推动经济发展由要素驱动向创新驱动转变;着力统筹城乡发展，推动社会结构由城乡二元向城乡一体化转变;着力建设资源节约型、环境友好型社会，推动我市由传统工业城市向现代生态城市转变，以奋发向上、大有作为的精神状态推动唐山科学发展、争先进位，为“十二五”开好局、起好步，以优异成绩向建党90周年献礼。全市国民经济和社会发展的主要预期目标是：地区生产总值增长11%;全部财政收入增长12%，其中一般预算收入增长13%;城镇居民人均可支配收入增长11%，农民人均纯收入增长11%;全社会固定资产投资增长18%;万元生产总值能耗、主要污染物排放量进一步下降，完成省达目标要求;城镇登记失业率控制在4.5%以内;人口自然增长率控制在4.91‰以内。

完成今年的各项目标任务，加快经济发展方式转变，实现经济社会又好又快发展，我们要坚持统筹兼顾，大力实施“两极三带”发展战略，以曹妃甸新区和市主城区两个增长极的率先突破，带动沿海经济带、平原经济带、山前经济带协调发展，活跃全市经济社会发展全局。重点做好十个方面的工作：

(一)大力推进产业结构调整，加快构建具有唐山特色的现代产业格局。围绕推进新型工业化，坚持以项目为抓手，以信息化和工业化融合为着力点，注重改造提升传统优势产业与发展战略性新兴产业并重，调强存量、调优增量，全面优化产业产品结构，努力保持经济持续平稳较快增长。

改造提升传统优势产业。加快运用高新技术和先进适用技术改造提升钢铁、装备制造、建材、化工、能源等传统产业。深入推进钢铁产业向精深加工和产业链高端发展，大力发展耗钢产业，加快渤海、长城钢铁集团深度整合，实施津西150万吨热轧钢板桩、首钢迁钢无取向硅钢等280个项目，年内精品钢比重达到63%，打造精品钢生产基地。以冀东发展、陶瓷集团等企业为龙头，推进水泥、陶瓷等产业整合和技术改造，打造北方重要的新型建材基地。以曹妃甸工业区、南堡开发区、乐亭新区为依托，加快化工产业发展，打造全国重要的现代化工临港产业基地。

加快发展先进装备制造业。以工程装备、自动焊接设备、冶金装备、修造船为重点，以开平现代装备制造园和曹妃甸装备制造园等园区为依托，着力抓好盾石机械搬迁改造、华电曹妃甸重工等一批重点项目建设，培育壮大一批优势产品、骨干企业和产业集群，加快把唐山建成中国北方重要的先进装备制造业基地。

培育发展战略性新兴产业。加强规划引导和政策扶持，鼓励传统优势产业资本特别是民营资本向战略性新兴产业转移。引进和培育新一代信息技术、节能环保、新能源、高端装备制造、新材料等产业项目，着力发展壮大高速动车组、中低速磁悬浮轨道客车等高端装备制造业，加快矿用抢险探测机器人、新能源汽车等产业化进程，谋划实施一批海洋工程装备项目，积极推进太阳能光伏光热发电、海上风力发电、生物质能发电和再生资源利用。全年战略性新兴产业投资增长20%以上。

大力发展服务业。坚持把生产性服务业摆在优先发展的位置，尤其是加快发展现代物流业，全力推进曹妃甸物流产业聚集区等大型综合性物流园区和物流重点项目建设。积极引导工业企业主辅分离，加快推进服务环节外包、剥离和转型。着力发展金融、保险、科技研发、信息咨询、市场营销等现代服务业。充分发展商贸流通等传统服务业，加快建设大型零售、星级酒店、特色商业街区等一批商业设施。以唐山湾国际旅游岛、清东陵为龙头，以历史文化旅游、生态观光旅游、蓝色海洋旅游为重点，以建设完善北部山前和南部沿海大交通网络为支撑，推动旅游业大发展、快发展。进一步优化服务业发展环境，在土地等要素配置上给予倾斜。全年服务业增加值增长13%以上。

加快民营经济和中小企业发展。加大政策扶持力度，创优发展环境，激活民间资本，放心、放开、放宽、放胆、放手推动民营经济快发展、大发展。鼓励个体工商、股份制、三资等各类所有制企业共同发展，鼓励大中小各类规模企业竞相发展，鼓励一二三产业各个行业的企业融合发展，鼓励民营企业参与国有企业改制重组、转型发展，鼓励民间投资发展第三产业和新兴战略产业。

加强重点项目和工业聚集区建设。全年谋划建设重点项目1000个以上，确保300个项目开工建设，100个项目完工投产。着力破解土地、资金等瓶颈制约，完善促进项目落地的体制机制。加快丰润中国动车城、开平现代装备制造园等重点工业聚集区建设，为项目加速聚集创造条件。年内每个工业聚集区新开工建设重点工业项目5个以上，工业项目投资完成10亿元以上，主营业务收入超200亿元的工业聚集区达到5个以上。

(二)加快唐山湾“四点一带”开发建设步伐，打造沿海经济隆起带。抓住河北加快沿海开发机遇，确定今年为我市加快唐山湾发展攻坚年。实施沿海倍增计划，唐山湾“四点一带”地区完成固定资产投资占到全市50%以上，其中曹妃甸新区完成投资1000亿元以上。

完善基础设施。加快港口建设，拓展港口物流保税服务功能，积极推进曹妃甸综合保税港区申报工作，唐山港货物吞吐量力争达到2.7亿吨。进一步加快水、电、路、讯等基础设施配套，力促京唐城际高铁早日获得国家批准，抓好滨海公路唐山段、海滨大道西连(天津)东扩(秦皇岛)等重点交通项目建设，积极推进迁曹高速、工业区与新城轨道交通等项目前期工作，构筑融入京津、贯通秦唐沧、连接环渤海的现代化交通体系。

加速临港产业聚集。按照唐山湾各园区产业定位，加快精品钢铁、化学工业、装备制造、现代物流和高新技术等主导产业发展，抓好华海风力发电装备制造、上汽电动汽车及锂源锂离子动力电池等一批重点项目建设，推动大型石油炼化一体化、首钢京唐钢铁二期等重大项目前期工作，加快矿石、煤炭、钢铁和综合四大物流园区建设，积极培育高新技术和战略性新兴产业。加快中日唐山曹妃甸生态工业园建设，在高标准编制各项规划基础上，加快基础设施配套，加大宣传推介力度，确保一批项目落地。

加快唐山湾生态城建设。以科教城区和企业服务区、滨海旅游区为重点，实施基础设施、招商引资、商业服务设施建设三大战役，加快完善商务、居住、医疗、教育等生活配套体系。突出抓好欧洲风情小镇、可持续发展中心、曹妃甸论坛会址等项目建设，尽快形成功能完善的城市组团，为聚集人口和产业创造条件。

推动沿海与内地互动。制定鼓励承接京津及周边地区产业项目转移的政策措施，启动冀东北工业聚集区、北京产业园、内蒙古临港产业园等园区建设，拓宽港口和港区发展腹地，引导生产力布局向沿海转移，实现沿海与腹地良性互动、协调发展。

(三)加大科技创新力度，为资源型城市转型和发展方式转变提供支撑。着眼于提升城市核心竞争力，把科技创新摆在更加突出位置，推动经济社会发展尽快走上创新驱动、内生增长的轨道。今年市财政安排科技专项资金1.7亿元，比上年增长22.9%。

加快建设创新平台、构建区域创新体系。以建设国家创新型试点城市为契机，以提高自主创新能力、破解经济社会发展中的科技瓶颈为目标，加快建立以企业为主体、市场为导向、产学研相结合的技术创新体系。充分发挥唐山国家高新技术开发区和中科院唐山研究转化中心的示范引领作用，围绕搭建科技创新研发、合作、服务、转化平台，突出推进电动汽车、循环经济、高速动车组等十大科技研发中心建设，新培育建设市级以上重点实验室、工程技术研究中心等各类技术研发机构20家。

加大重点产业关键核心技术引进推广力度。根据经济社会发展重大关键共性技术需求，实施一批重大科技专项。在加强原始创新、集成创新的同时，着力开展引进消化吸收再创新。围绕做优做强精品钢铁、先进装备制造、现代化工等重点产业，研究开发和引进示范一批新技术新工艺，加快科技成果转化。全年转化实施重大专利技术150项，申请专利、授权专利比上年提高15%。

全面提升企业自主创新能力。充分发挥本地科技、教育资源作用，推行企业技术创新计划，加大政策扶持力度，引导企业增加科技创新投入，鼓励企业引进新技术、开发新产品，支持企业与高校、科研院所联合建立研发机构。积极培育科技型中小企业，加快建设拥有自主知识产权、核心技术、知名品牌和具有国际竞争力的创新型企业。

大力引进培养创新型人才。加快实施人才强市战略，落实相关政策，优化人才环境，全面做好人才引进、培养、使用工作。特别是加大对高层次急需人才、领军人才和研发团队、高技能人才和国际化人才智力的引进力度，推进“院士联谊”“假日博士”等借智工作，建立海外高层次人才创新创业基地。根据我市经济社会发展实际需要，全年新增特邀院士10人以上，引进、培养博士100人以上、硕士1000人以上。

(四)强力推进生态建设，加快构建资源节约型、环境友好型社会。牢固树立绿色发展、低碳发展理念，综合运用经济、法律和必要的行政手段，毫不放松地抓好生态建设各个领域、各个环节工作，提高生态文明建设水平。

强力推进节能减排和淘汰落后产能。严格落实节能减排责任制，加大考核奖惩力度，推进节能减排工作常态化、制度化。开展节能减排对标行动，突出抓好300家能耗在5000吨标准煤以上企业的能效对标，强化对156家重点污染减排企业的对标核查，实施国丰钢铁烧结机余热联合发电等69个节能项目和首钢京唐钢铁烧结机脱硫等62个工业减排项目，在钢铁、焦化、建材等8个行业推广一批节约、替代、循环利用和治理污染的先进适用技术。加大落后产能淘汰力度，年底前全市淘汰400立方米及以下炼铁高炉和30吨及以下炼钢转炉、电炉。强化行业准入管理，严控高耗能、高污染企业盲目发展。

加强环境保护和生态修复治理。继续实施滦河、陡河、还乡河等重点流域的水污染治理工程，加强水源地保护。加大农村生态环境整治力度，支持鼓励乡镇建设生活垃圾无害化处理设施。严格污染物排放标准和环境影响评价，强化执法监督，健全重大环境事件和污染事故责任追究制度，切实解决好影响群众切身利益的突出环境问题。加强水资源管理，科学有序开发矿产资源。开展低碳生活“进家庭、进社区、进校园、进车间”行动，加快构建资源节约、环境友好的产业结构、增长方式和消费模式。

大力发展循环经济。抓好曹妃甸国家级循环经济示范区和司家营省级循环经济示范园区建设，推进三友盐化工等20个循环经济示范项目和迁安大中型沼气等26个低碳经济发展项目。制定实施重点污染行业、企业及单位的清洁生产审核计划，鼓励支持工业“三废”综合利用和再制造产品生产，完善再生资源回收体系和垃圾分类回收制度。推进生产、流通、消费各环节以及企业、区域和工业、农业、城市各领域循环发展。

(五)以推进农业现代化为重点，切实做好“三农”工作。坚持统筹城乡发展，加大投入力度，加快社会主义新农村建设，促进农业增效、农民增收、农村繁荣。

加快发展现代农业。深入推进农业结构战略性调整，实施龙头企业壮大、品牌农业创新、粮食核心区建设、农田水利建设等16项重点工程，加大农业项目扶持力度，提高农业专业化、标准化、规模化、集约化水平。加快各县(市)区现代农业示范园区和农产品加工园区建设，新发展国家级农业龙头企业2家、省级10家、市级50家，新发展农民专业合作社150个以上，农业产业化经营率达到65%。

加快推进城乡等值化、一体化进程。深入推进统筹城乡发展试点工作，着力在城乡规划、产业布局、基础设施建设、劳动就业与社会保障、公共服务、社会管理一体化方面取得突破。尤其是积极推进农村电网、交通等基础设施建设和各项社会事业发展，逐步实现城乡基本公共服务均等化。妥善解决好失地农民、失海渔民的生活安置和社会保障问题。深化户籍制度改革，促进农民向城镇有序转移。

加快农业农村体制机制创新。完善土地承包经营权流转市场，加大财政奖补政策扶持力度，发展多种形式的适度规模经营。深化农村产权制度改革，基本完成各项产权确权、登记、颁证工作，积极发展新型集体经济。优化农村金融环境，市县两级各建立担保资金不少于2亿元和5000万元的农业担保公司，每个县(市)区发展5个以上农村资金互助合作社。

加快农村新民居建设。坚持政策引导、因地制宜、分类指导，充分尊重农民意愿，实施350个省级新民居示范村建设。重点抓好20个市级联村(中心村)建设，培育40个新民居精品示范典型。注重把新民居建设与发展生态农业、休闲观光旅游等产业结合起来，与开发传统文化、传承“民俗文化”结合起来，促进农民增收致富。深入实施农村清洁工程，开展绿色家园创建活动，进一步改善农村生产生活环境。

(六)以承办世界园艺博览会为契机，全面提升城市建设管理水平。着眼打造宜居宜业的现代化生态城市，加快新型城镇化进程，巩固扩大城镇面貌三年大变样成果,推动城镇建设三年上水平,坚持外延拓展与旧城区改造相结合、提升外在形象与聚集产业相结合、建设与管理相结合，加大城镇改造建设力度，增强城市产业聚集力、经济辐射力、人口承载力和综合竞争力。今年城镇建设力争投资1000亿元。

围绕构筑现代化新城区，推动南湖生态城、唐山湾生态城、凤凰新城、空港城、环城水系和唐山湾国际旅游岛开发建设“六个提速”。南湖生态城，以建成国家5A级景区为目标，年内完成南湖景观大道、访客中心、西北片区路网等基础设施建设，加快实施老唐山风情小镇、市民中心、文化广场等公建设施建设，进一步完善功能、提升景观。唐山湾生态城，以建设绿色生态城市为目标，加快造地、路网、市政、生活配套设施等工程建设，完成唐山工业职业技术学院新校区、可持续发展中心、起步区道路网格化等一批重点工程建设。凤凰新城，进一步完善路网和市政配套设施，加快科技馆、总部基地、香格里拉大酒店、传媒大厦等重点项目建设。空港城，抓好路网和污水处理等基础设施建设，尽快拉开开发建设框架。环城水系，实施水系周边大城山、弯道山、凤山、青龙湖、大洪桥、河北桥区域等六大片区改造，打造环城产业发展带、生态景观带。唐山湾国际旅游岛，完成旅游专用线、码头等项目建设，启动实施温泉度假区、祥云岛海上影视城等项目开发，加快打造独具特色的国际旅游岛。同时，科学有序推进2016年世界园艺博览会各项筹备工作，制定五年筹备工作计划，年内完成规划设计，开展园址生态绿化，启动招展工作。

围绕加快旧城区改造提升，全力抓好“六大改造”工程。一是加快震后危旧平房改造，力争市区229万平方米安置住房主体完工。二是启动凤山区域改造，结合环城水系开发，建设集休闲度假、居住旅游、文化运动为一体的多功能郊野生态风景区。三是启动新火车站区域综合改造，发展高端商业、服务业，打造现代化新城区。四是加快弯道山区域综合改造，建设以陶瓷文化展示、商务办公、贸易物流、居住休闲为主的综合性城市新区。五是启动大城山区域改造，建设唐人文化园，打造体现唐山根源文化的新城区。六是加快市区城中村改造步伐，年内市区建成区城中村改造全面完成，南湖西北片区回迁房主体完工，外环线周边村庄改造全部启动。

围绕提升城市形象、加速产业和人口向城镇聚集，集中力量组织开展“六项攻坚战役”。一是拆违拆迁攻坚，年内完成400万平方米以上。二是园林绿化攻坚，建成区绿地率、绿化覆盖率分别提高1个百分点以上。三是基础设施建设攻坚，新建、扩宽、改造15条城市主次干道，加快西郊、北郊、南郊三个热电厂建设。四是标志性建筑攻坚，确保新火车站、新青少年宫等20个标志性建筑主体工程竣工。五是市容环境整治攻坚，对城市主要街道两侧既有建筑实施景观改造、亮化升级。六是县城扩容攻坚，实现县(市)城控制性详规全覆盖，每个县(市)扩容1平方公里以上，50%以上城中村启动建设。以重点镇为龙头，加快小城镇建设步伐，增强承载和辐射带动能力。

(七)统筹经济社会协调发展，促进各项社会事业全面进步。在发展经济的同时，更加重视教育、文化、卫生、体育等社会建设和发展。今年全市财政投入社会事业资金增长43.1%。

优先发展教育事业。全面优化基础教育，高度重视学前教育，加强公办幼儿园建设，鼓励民办幼儿园发展，扎实推动义务教育均衡发展，率先在全省高水平通过普及高中阶段教育评估。完善“以县为主”的义务教育投入保障机制，不断增加教育投入。着力改善城乡中小学办学条件，加快实施中小学校舍安全工程，力争全面完成改造任务。大力发展职业教育，将未升入大学和高中的学生全部纳入职业教育范围，抓好城镇企业职工和下岗再就业人员技能培训，实施“百万农民大培训”、新农村建设“双带头人”培训等工程，努力使人人拥有一技之能。加快提升高等教育水平，加强高校重点学科和博士点、硕士点建设，扩大与国内外知名高校的交流与合作，谋划推进高等教育资源整合。鼓励发展民办教育。健全继续教育体系，加强从业人员继续教育和培训，促进学习型社会建设。加强师德师风建设和教师业务培训，提高教师队伍整体素质。加大对贫困家庭学生的救助力度，保障农民工子女、农村留守儿童等受教育权利。

推进和谐文化建设。加强社会主义核心价值体系建设和理想信念教育，大力弘扬“感恩、博爱、开放、超越”的新唐山人文精神，汇聚推进唐山科学发展、争先进位的强大精神力量。以全国文明城市、国家卫生城市和全国健康城市“三城联创”为载体，广泛开展群众性精神文明创建活动，提高城市文明水平。繁荣发展文化事业，加快博物馆、图书馆、群艺馆、文化馆等公益性文化设施建设，推进乡镇综合文化站和“农家书屋”等文化惠民工程，大力发展社区文化，活跃群众文化生活。加快发展文化产业，重点培育现代传媒、演艺文化等七大文化产业业态，加快唐人文化园等十大特色园区和中国近代工业博物馆、文化广场等文化重点项目建设，提升文化产业综合竞争力。

加快医疗卫生事业改革发展。继续深入开展“健康唐山、幸福人民”行动，实施健康教育、健康服务、健康饮食、健康文体、健康环境、健康单位、健康生活、健康社会、健康信息九大工程，扎实推进健康城市建设，年内为全市45岁以上农民、城镇无业居民中的“三高”人群进行免费体检。深化医药卫生体制改革，加快推进基本医疗保障制度建设，积极建立国家基本药物制度和基本医疗卫生机构补偿机制，稳步开展公立医院改革试点，鼓励社会办医。完善城镇居民医疗保险和新型农村合作医疗制度，新农合参保率达到96%以上。以农村和城市社区为重点，进一步健全基层医疗卫生服务体系。加强卫生基础设施建设，进一步改善群众就医条件。健全完善公共卫生体系，抓好麻疹等传染病预防控制工作。

全面发展体育、人口等各项事业。认真贯彻国家《全民健身条例》，广泛开展全民健身运动，提高竞技体育水平，大力发展体育产业，着力建设好“南湖国家体育休闲示范区”，推进奥体中心等基础设施建设。认真落实计划生育基本国策，加强依法管理、利益引导和优质服务，继续稳定低生育水平，提高出生人口素质，促进出生性别比平衡。加强未成年人保护，保障妇女儿童权益。加快社会养老机构建设，促进社会养老事业发展。全面贯彻党的民族宗教政策，维护民族团结，促进宗教和谐。协调发展广播电视、外事侨务、国防动员、人民防空、气象、防震减灾、档案等各项事业，促进社会全面进步。

(八)全力保障和改善民生，不断提高人民群众幸福指数。围绕解决人民群众最关心、最直接、最现实的利益问题，今年拟投入160亿元，重点为群众办好20件实事，涉及就业、社会保障、居住条件改善、交通出行、医疗卫生、文化休闲、市场建设等方面。就业方面，实施更加积极的就业政策，多渠道增加就业岗位，年内城镇新增就业10万人以上，向非农领域转移农村富余劳动力15万人，推荐1万个高校毕业生就业见习岗位。社会保障方面，扩大新型农村社会养老保险覆盖范围，为农村60万人办理新型社会养老保险，全市农村适龄人口参保人数由120万人增加到180万人;再次提高城乡低保标准，城镇每人每月由310元提高到363元，农村每人每年由1540元提高到1900元。居住条件改善方面，为市中心区中低收入家庭提供保障性住房28万平方米;市区震后危旧平房改造全部开工，建设安置住房203万平方米;市区新增天然气用户1万户，集中供热用户1.2万户。交通出行方面，改造一批城市道路，新建改造农村公路743公里、改造维修桥梁56座;市区新增200部公交车;完成唐山新火车站站房改造主体工程，年内部分投入使用;唐山三女河机场新开通航线6条、达到10条。医疗卫生方面，加快建设凤凰新城新工人医院，开工建设新妇幼医院，努力解决群众看病难问题。文化休闲方面，完成唐山博物馆改造扩建并免费向市民开放，市植物园部分向市民开放;开工建设中国近代工业博物馆、唐人文化园、新工人文化宫、文化广场、奥体中心、新图书馆和野生动物园。市场建设方面，市区新建、改造城市标准化菜市场30个,方便群众生活。同时，按照中央、省部署，实施“米袋子”“菜篮子”工程，以保障基本农产品市场供给、保障困难群体基本生活为重点，加强市场价格预警监管，下大力整顿和规范市场价格秩序，确保市场价格总水平基本稳定。

(九)加快推进各项改革，全方位扩大对内对外开放。以更大的决心和力度深化改革、扩大开放，为转变经济发展方式、实现又好又快发展提供强大动力、注入新的活力。

深化重点领域和关键环节改革。深化行政体制改革，在全面完成市县政府机构改革的基础上，按照省统一部署，积极推进乡镇机构改革。深化审批制度改革，继续削减和调整行政审批事项，加强电子政务建设。积极稳妥推进科技、教育、文化等事业单位分类改革。深化国有企业改革，支持困难企业改制发展，加速优势企业战略重组，建立健全现代企业制度。深化财政领域改革，优化财政支出结构，健全公共财政体制;依法严格税费征管，大力压缩一般性支出，加强政府债务管理，确保财政稳定增长、平稳运行。深化金融体制改革，加快市商业银行战略重组、农村信用社改革和曹妃甸农村商业银行、村镇银行组建，抓好凤凰新城金融区建设，积极推进设立地方金融控股集团、创业投资基金、融资担保等各类新型金融业态，努力构建冀东区域金融中心。

加快构筑全方位开放新格局。进一步强化开放意识，在注重引进资金、项目的同时，全面引进技术、管理、人才、品牌等先进生产要素。创新招商方式，突出招商重点，巩固扩大与日本、韩国、新加坡的合作，积极拓展与欧洲、北美的联系，全面深化与港澳台的经贸往来，进一步提高招商实效。重点推进60个千万美元以上外资项目签约、50个重点外资项目履约开工。全力办好第二届曹妃甸论坛和第二届中国•曹妃甸临港产业国际投资贸易洽谈会，确保收到实效。全年实际利用外资11.2亿美元，增长27%。优化出口产品结构，做大做强出口企业和出口基地，全市出口总额增长13%。加快实施“走出去”战略，支持有条件的企业开展境外投资，推动钢铁、建材等优势行业产能转移，全年新增对外投资项目10个以上。全面加强与京津及长三角、珠三角地区的合作，全年引进市外资金370亿元。

(十)积极推进社会管理创新，全力维护社会稳定。适应经济体制、社会结构深刻变化的新形势，进一步加强社会管理法制、体制、能力建设，加快健全党委领导、政府负责、社会协调、公众参与的社会管理新格局，促进社会治理和谐化。

深入实施社会矛盾综合调控，更加注重社会矛盾源头预防治理，进一步建立健全利益协调、诉求表述、矛盾调处和权益保障机制，完善重大决策社会稳定风险评估机制和社会稳定指数评价体系，严格执法监督和公正廉洁执法，维护社会公平正义。怀着深厚感情认真做好群众来信来访工作，正确处理人民内部矛盾，切实解决好关系群众切身利益的突出问题。建立健全行政调解工作机制和制度。拓展城乡一体化服务管理信息系统服务内容和领域，不断提高政府公共服务能力。加强社会治安综合治理，深入推进依法治市，广泛开展普法教育，继续实施“平安唐山”创建工程，完善社会治安科技防范体系，依法严厉打击各类违法犯罪活动，努力提高人民群众的安全感。健全各种事故灾难、公共卫生事件、食品安全事件、社会事件预防预警和应急处置体系，提高应对各种突发公共安全事件的能力。完善基层管理和服务体系，形成社会管理和服务合力。

加强安全生产和食品药品安全工作是事关人民群众生命财产安全的大事，是各级政府义不容辞的重大责任，必须毫不放松地抓紧抓实抓好。健全完善监督管理体制，明确地方和部门监管责任，持续开展专项整治，建立监管长效机制，确保不发生重特大安全生产和食品药品安全事故。

三、关于《国民经济和社会发展第十二个五年规划纲要(草案)》的说明

当前，我们已进入“十二五”新的发展时期。科学制定和有效实施“十二五”规划，对于推进唐山科学发展，实现新的跨越，具有十分重要的意义。市委八届七次全会通过的《关于制定国民经济和社会发展第十二个五年规划的建议》，提出了未来五年我市国民经济和社会发展的奋斗目标、指导方针和主要任务。根据《建议》精神，市政府制定了《国民经济和社会发展第十二个五年规划纲要(草案)》，提请大会审议。下面，就几个问题作简要说明。

(一)《纲要(草案)》的编制过程和主要特点

市委、市政府对制定“十二五”规划高度重视。早在2009年9月就开始部署相关准备工作，先后提出了39个重大课题和36个专项规划，落实到市直相关单位和部门分别进行研究。同时，市政府还委托国家行政学院对“十二五”期间我市经济社会发展的基本思路进行了专题研究，为编制《纲要(草案)》提供了基础。

市委八届七次全会召开以后，市政府根据市委《关于制定国民经济和社会发展第十二个五年规划的建议》精神，深入研究和编制《纲要(草案)》，并召开政府常务会议对《纲要(草案)》进行了专题讨论。在编制过程中，充分吸纳了“我为‘十二五’规划献一计”和全市“转变经济发展方式，调整经济结构，推动科学发展”调研月活动成果，广泛征求了各县(市)区、各部门、各人民团体的意见，认真听取了各民主党派、工商联和无党派人士的意见和建议。可以说，《纲要》的形成过程，是认真总结过去、全面规划未来的过程，是发扬民主、科学决策的过程，是统一思想、形成共识的过程。

《纲要(草案)》力求体现在新时期、新形势下推进唐山转变发展方式、加快资源型城市转型、实现科学发展的要求，体现宏观性、战略性和政策性。在规划指标上，提出了确保目标和力争目标两套指标。确保目标是在全面分析未来五年经济社会发展面临的有利条件和不利因素的基础上提出的，是必须确保完成的。力争目标是在确保目标的基础上，围绕推进唐山科学发展、争先进位，对全市经济社会发展提出的更高要求，以此鼓舞和激发全市上下奋发有为地推动唐山实现跨越式发展。

(二)“十一五”期间国民经济和社会发展主要成就

“十一五”时期是我市发展史上极不平凡的五年。五年来特别是党的十七大以来，全市上下以贯彻落实胡锦涛总书记视察唐山重要指示精神为动力，深入贯彻科学发展观，积极应对国际金融危机冲击，创造性地推动各项工作，经济社会发展取得了重大成就，圆满完成了“十一五”规划的各项目标任务。

五年来，我市综合实力大幅度提升。全市生产总值登上4000亿元台阶，为2005年的1.9倍,“十一五”年均增长13.4%;全社会固定资产投资比“十五”增长3倍;全部财政收入、一般预算收入分别为2005年的1.9倍和2.5倍;一般预算收入占全部财政收入比重达到44.6%，比“十五”末提高10.3个百分点，地方可用财力净增加118.4亿元。经济总量和财政收入均保持全省第一位。

五年来，我市经济结构调整取得明显成效。农业综合生产能力和农业产业化经营水平稳步提高，全市农业产业化经营率比“十五”末提高9个百分点。工业结构调整步伐加快，传统产业改造升级、战略性新兴产业和现代服务业发展都取得突破性进展，发展的质量效益和规模档次实现了新的提升。

五年来，我市生产力布局进一步优化。以曹妃甸新区为龙头的唐山湾“四点一带”开发建设加速推进。“四点一带”区域经济总量、财政收入、固定资产投资分别占全市的32%、26%和52%。特别是曹妃甸新区发展势头强劲，累计完成投资2500多亿元，进入了产业加速聚集、城市配套发展的新阶段。

五年来，我市城乡面貌显著改观。“四城一河”开发建设取得重要进展，改变了唐山传统资源型城市的固有形态，城市框架迅速拉开，城市功能不断完善，城市形象和品位明显提升。城镇化率由46.1%提高到54.9%，城镇化发展指数位居全省首位。新农村建设扎实开展，城乡一体化水平大幅提升。

五年来，我市生态建设步伐不断加快。持续开展节能减排攻坚行动，“十一五”节能减排目标能够如期完成。组织开展“绿化唐山攻坚行动”，森林覆盖率由2005年的21.9%提高到28.7%。2010年城市空气质量二级及优于二级天数比2005年增加14天。

五年来，我市改革开放进一步深化。国有企业改革、行政管理体制改革、财税体制改革、投融资体制改革等各项改革深入推进，文化体制改革、医药卫生体制改革全面启动。对外开放不断扩大，五年累计实际利用外资37.1亿美元，是“十五”时期的2.6倍;2010年进出口总额为“十五”末的2.7倍。

五年来，我市和谐社会建设取得新进展。城乡居民收入大幅提高，城镇居民人均可支配收入、农民人均纯收入年均分别增长13.2%和12.6%。城镇就业累计新增34万人。社会保障体系不断完善。全市财政用于社会事业投入年均增长23.8%，高于经常性财政收入增幅8.8个百分点，科技、教育、文化、卫生、体育等各项社会事业全面发展，和谐稳定局面不断巩固。

可以说，“十一五”时期是我市经济社会发展最好最快、城乡面貌变化最大、人民群众得到实惠最多的时期之一，为“十二五”时期的发展奠定了坚实基础。成绩来之不易，经验弥足珍贵。

(三)“十二五”时期经济社会发展的指导思想和奋斗目标

“十二五”时期是我市科学发展的关键时期，是深化改革开放、加快转变经济发展方式的攻坚时期。综合分析各种因素，未来五年我市经济社会发展仍处在重要战略机遇期和黄金发展期。我们必须牢牢抓住机遇，时不我待地推动唐山又好又快发展，赢得未来发展的主动权。

《纲要(草案)牘》全面贯彻落实科学发展观，体现了中央和省委、市委《建议》提出的制定“十二五”规划的指导思想和做好“十二五”时期工作的总体思路要求。在“十二五”期间，我们要牢牢把握奋发向上、大有作为这一基调，紧紧围绕实现科学发展、争先进位这一目标，突出加快转变经济发展方式这一主线，加快实施科教立市、以港兴市、人才强市三大战略，强力推进生态城市建设，以重大产业项目为抓手，以产业聚集区建设为载体，构建具有唐山特色的现代产业格局;以“两极”(曹妃甸新区增长极、市主城区增长极)率先突破，促进“三带”(沿海经济带、平原经济带、山前经济带)跨越发展;加快结构调整，深化改革开放，切实保障和改善民生，实现由经济大市向经济强市转变，加快把我市建设成为东北亚地区经济合作的窗口城市、环渤海地区新型工业化基地和首都经济圈的重要支点,建设成为科学发展示范区和人民群众幸福之都。在工作中，要正确把握和处理好“十个方面”关系，即：把握和处理好保持经济平稳较快发展与转变经济发展方式的关系、优化提升传统产业与培育发展战略性新兴产业的关系、发展沿海经济与加快沿海工业聚集区建设的关系、节能减排与产业结构调整的关系、城镇建设与发展第三产业的关系、农业现代化与农业产业化的关系、科技创新与扩大开放的关系、深化改革与发挥市场机制作用的关系、保障改善民生与做好群众工作的关系、转变干部作风与完善干部考核评价机制的关系，努力开创唐山科学发展新局面。

根据市委《建议》，《纲要(草案)》提出了我市“十二五”时期经济社会发展的主要目标，包括六大方面，即：综合实力显著增强，经济结构优化升级，改革开放不断深化，科技创新实现突破，资源环境明显改善，社会建设全面加强。“十二五”规划目标的制定，力求体现推进唐山加快转变发展方式和资源型城市转型、实现科学发展的要求，体现惠及民生、和谐发展的要求。这里，仅就经济增长速度指标作简要说明。

《纲要(草案)》提出，“十二五”时期全市主要经济指标显著提升，确保地区生产总值、全部财政收入、财政一般预算收入年均分别增长11%、12%、13%，力争分别增长12.5%、15.9%、19%，占全省的比重分别提高到25%、23%和20%，达到或基本达到全省四分之一的目标，这既是省委、省政府对唐山的要求和期望，也充分考虑了我市现阶段的发展实际和现实基础，是促进唐山科学发展、实现发展速度和发展质量双提升的客观需要。我们要经过“十二五”的大发展，使全市综合实力实现大的提升，经济社会发展实现大的跨越，人民生活实现大的改善。这一目标的确定，无论从我市扩大就业、增加收入、维护稳定的要求看，还是从经济增长潜力、内外部发展环境看，既是积极稳妥的，也是切实可行的。

(四)“十二五”时期的战略重点和主要任务

《纲要(草案)》对“十二五”时期的经济建设、社会发展、改革开放等方面都作了全面部署，提出了明确的任务和措施。

1.以“两极”率先突破，促进“三带”跨越发展。统筹沿海与内陆发展，打造曹妃甸新区增长极和市主城区增长极，加快沿海经济带开发开放、平原经济带产业升级和山前经济带转型发展，形成以“两极”率先突破促“三带”融合发展的新格局。曹妃甸新区和市主城区两大增长极，分别在主导产业聚集上和调整经济结构上率先取得突破;沿海经济带倍增，加快开发开放，着力打造唐山科学发展、争先进位的引擎;平原经济带进位，加快产业升级，建设沿海与内陆互动的平台;山前经济带争先，加快转型发展，形成具有地域特色、功能完备的区域经济。

2.转变经济发展方式，调整优化经济结构。运用先进适用技术改造提升传统产业,以信息化带动工业化，突出发展先进装备制造业，做优做强钢铁产业，延伸发展化工产业，调整优化建材等优势产业。培育壮大新能源、生物医药、节能环保、电子信息、新材料等战略性新兴产业，加快形成先导性、支柱性产业。以生产性服务业为重点，大力发展服务业。巩固农业基础地位，加快农业现代化进程。发展能源等基础产业，加快交通等基础设施建设，增强经济发展保障和支撑能力。大力发展民营经济，促进大中小企业协调发展，培育新的经济增长点。

3.加快推进城镇化，建设生态宜居现代化城市。优化城镇布局，强化市主城区和唐山湾生态城“双核”的辐射带动作用，协调推进城镇建设，构建城镇发展新格局。加快旧城改造步伐，推进“四城一河三岛”等重点区域开发，不断拓展城市发展空间。加强城市路网、市政设施等基础设施建设，提升城市承载力。完善城市服务功能，健全城市管理体制，创新管理手段，加快数字化城市管理系统建设，提升城市建设管理水平。

4.统筹城乡发展，建设社会主义新农村。大力发展县域经济，着力培育发展区域特色主导产业，做大县域经济总量，提升发展层次。加快推进城乡规划、产业、基础设施建设、就业和社会保障、公共服务和社会管理一体化，创新公共财政、公共服务、公共管理、公共政策保障机制，促进城乡一体化发展。深化农村土地管理、产权制度、金融、户籍等综合配套改革，激发农村发展活力。

5.提高科技创新和人才支撑能力，建设创新型城市。充分发挥企业主体作用，加大政府创新投入，实施技术创新工程，加快创新体系建设。完善创新创业融资环境，大力发展各类科技中介机构，打造创新服务平台。以产业优化升级为目标，加快技术创新平台建设，全面推进产业技术进步。深入实施人才强市战略，着力引进和培养高层次创新创业人才，营造有利于人尽其才、才尽其用的社会氛围。

6.加强生态文明建设，增强经济可持续发展能力。强力推进节能减排，深入开展节能减排对标行动，加强节能减排工程项目建设，完善节能减排政策激励约束机制，严控高耗能高污染行业过快增长。推进循环经济示范园区和示范企业建设，全面推行清洁生产，大力发展循环经济和低碳经济，不断提高资源综合利用水平。切实加强自然资源和生态环境保护，形成节约型生产方式、交通方式和消费方式。

7.深化改革开放，增强经济社会发展的活力和动力。加快行政管理体制、财税和金融体制、国有企业和资源性产品价格等重点领域改革步伐，努力破解制约发展的体制机制障碍。不断扩大利用外资规模，提高利用外资质量。优化出口产品结构，积极推动市场多元化，促进对外贸易大发展。大力实施“走出去”战略，在更高层次上参与国际分工与合作，拓展海外发展空间。加强区域经济合作，形成协调发展、互补发展的区域发展新格局。

8.加快文化建设步伐，促进文化大发展大繁荣。加强社会主义核心价值体系建设和理想信念教育，广泛开展群众性精神文明创建活动，提高全民文明素质。大力发展文化产业，培育文化市场，推动我市由文化大市向文化强市转变。加大财政对公益性文化事业的投入，完善公共文化服务体系，发展优秀地域文化，切实繁荣文化事业。

9.坚持民生优先，全面构建社会主义和谐社会。实施扩大就业工程，千方百计扩大就业，不断增加城乡居民收入。坚持广覆盖、保基本、多层次、可持续的方针，健全社会保障和社会救助体系。加大保障性住房建设力度，构建以中低收入家庭为保障对象的多层次住房保障体系。优先发展教育事业，加快推进区域教育现代化进程，建设人力资源强市。深化医药卫生体制改革，加快发展医疗卫生事业，提高人民群众健康水平。加快发展体育事业和体育产业。全面做好人口工作。加快发展民政事业。推进社会主义民主法制建设，加强社会管理，维护社会安全稳定。

各位代表，全面完成今年和“十二五”期间的任务，对政府工作提出了新的更高要求，必须大力加强政府自身建设。

我们要坚持进一步解放思想，树立奋发向上、大有作为的精神状态。面对大有作为的重要战略机遇，勇于担当、敢于超越，自我加压、负重前进，以饱满的热情、坚韧的毅力、科学的态度，积极应对各种困难和挑战，创造性地推进各项事业发展。进一步强化责任意识、危机意识和竞争意识，在全国、全省大局中找准位置，明确目标，紧紧扭住发展这个第一要务，聚精会神搞建设，一心一意谋发展，既千方百计加快发展速度，又加快转变发展方式，推进唐山又好又快发展。

我们要坚持依法行政，不断提高政府公信力。健全落实重大问题集体决策、研究咨询、社会公示和社会听证等制度，促进科学决策、民主决策、依法决策。自觉接受人大及其常委会的法律监督、工作监督和政协的民主监督，认真办理人大代表、政协委员的批评、意见、建议和提案。诚恳听取各民主党派、工商联和无党派人士的意见和建议，自觉接受社会各界和群众监督。深入推进行政执法责任制，落实执法过错责任追究和执法行为评议考核制度，严格按照法定权限和程序行使权力、履行职责。

我们要坚持以人为本，尽心竭力为人民群众谋福祉。强化宗旨观念，把群众的要求作为第一信号，把群众的安危冷暖时刻挂在心上，真心实意为群众办实事、办好事。注重深入基层、深入群众，倾听群众呼声，体察群众疾苦，切实帮助基层群众解决实际困难和问题。认真研究解决好群众关心关注的就业、医疗、教育、住房等突出问题，高度关注困难群众的生产生活，让群众共享改革发展成果。

我们要坚持真抓实干，切实转变工作作风。坚持一切从实际出发，尊重科学、尊重规律，力戒官僚主义、形式主义，少说多做，真抓实干。严格落实工作责任制，健全完善考核奖惩机制，确保各项工作任务落到实处、收到实效。强化服务意识、效率观念，下大力解决行政不作为、乱作为问题，不断提升行政效能和服务水平，进一步优化发展软环境。大力倡导团结干事之风，讲大局、讲团结，在全市形成人心思进、团结奋进、开拓前进的生动局面。

我们要坚持廉洁从政，坚决预防和惩治腐败。深入推进政府系统惩治和预防腐败体系建设，认真落实党风廉政建设责任制，健全和落实“一岗双责”制度。进一步完善行政权力运行监控机制，切实加强监督管理，从源头上预防和减少腐败现象发生。大力倡导“两个务必”，坚决反对讲排场、摆阔气、挥霍奢侈、铺张浪费，以廉洁的政府形象取信于民。

各位代表，我们已经站在新的历史起点上，踏上了“十二五”新的发展征程。让我们更加紧密地团结在以胡锦涛同志为总书记的党中央周围，在省委省政府和市委的正确领导下，深入贯彻落实科学发展观，振奋精神，坚定信心，锐意进取，开拓创新，求真务实，埋头苦干，以奋发向上、大有作为的精神状态，实现唐山“十二五”科学发展、争先进位，为把唐山建设成为东北亚地区经济合作的窗口城市、环渤海地区新型工业化基地和首都经济圈的重要支点,建设成为科学发展示范区和人民群众幸福之都而努力奋斗!
